# The Relationship Between Metacognitive Beliefs and Salivary Cortisol, BDNF, and NDNF Levels: A Cross‐Sectional Study

**DOI:** 10.1002/brb3.71063

**Published:** 2025-12-10

**Authors:** Süheyb Okur, Bülent Bayraktar, Fatma Tosun Köse

**Affiliations:** ^1^ Faculty of Theology, Programme in Philosophy And Religious Studies, Department of Religious Psychology Bayburt University Bayburt Türkiye; ^2^ Faculty of Health Sciences Department of Physiotherapy and Rehabilitation Bayburt University Bayburt Türkiye; ^3^ Faculty of Theology Programme in Philosophy and Religious Sciences, Department of Philosophy of Religion Bayburt University Bayburt Türkiye

**Keywords:** BDNF, biomarker, cortisol, metacognition, NDNF

## Abstract

**Purpose:**

The main objective of this study is to non‐invasively investigate the relationship between metacognitive beliefs and cortisol, the primary stress output of the hypothalamic‐pituitary‐adrenal (HPA) axis, as well as neurotrophic factors associated with neuroplasticity brain‐derived neurotrophic factor and neuron‐derived neurotrophic factor (BDNF and NDNF). Within this framework, the hypotheses that negative metacognitions would be associated with increased cortisol and decreased BDNF levels, and that cortisol might play a mediating role in this relationship, were tested.

**Method:**

The study was designed in a cross‐sectional model with 60 university students. Participants' metacognitive beliefs were measured using the Metacognitions Questionnaire‐30 (MCQ‐30). Salivary cortisol, BDNF, and NDNF levels were analyzed using the ELISA method. Pearson correlation and hierarchical multiple regression analyses were used for data analysis.

**Finding:**

The results showed a significant positive relationship between the total metacognition score and cortisol (*r* = 0.589, *p* < 0.01) and a strong negative relationship between cortisol and BDNF (*r* = −0.662, *p* < 0.01). Hierarchical regression analysis supported a partial mediation model, indicating that dysfunctional metacognitive beliefs have both a significant direct negative association with BDNF and an indirect association mediated by cortisol. In the final model, both metacognition (*β* = –0.298, *p* < 0.05) and cortisol (*β* = –0.281, *p* < 0.05) were significant factors associated with lower BDNF levels. NDNF showed a positive relationship with BDNF (*r* = 0.571) but not with other variables.

**Conclusion:**

These findings point to a psychobiological model where dysfunctional metacognitive beliefs are linked to suppressed neuroprotective mechanisms like BDNF, both directly and indirectly through HPA axis activation. The results shed light on the potential neurobiological mechanisms underlying the effectiveness of metacognitive therapies.

## Graphical abstract

This study reveals that dysfunctional metacognitive beliefs are associated with lower brain‐derived neurotrophic factor (BDNF) levels through two pathways: a significant direct negative association and a significant indirect association mediated by increased cortisol levels, highlighting a key psychobiological mechanism of stress.

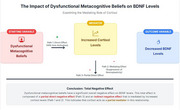

## 1 Background

In the twenty‐first century, chronic stress is recognized as a widespread public health problem playing a central role in the etiology of mood disorders such as anxiety and depression (Cohen et al. 2016). The physiological response to stress is largely managed by the hypothalamic‐pituitary‐adrenal (HPA) axis, and chronic activation of this axis leads to the sustained release of its end product, cortisol, at high levels (Herman et al. 2016; Smith and Vale 2006; Tsigos and Chrousos 2002). There is growing evidence that prolonged cortisol exposure leads to structural and functional impairments in brain regions vital for neuronal plasticity and cognitive functions, particularly the hippocampus and prefrontal cortex (Campbell and Macqueen 2004; Lupien et al. 2018). This condition reduces an individual's learning and memory capacity while increasing susceptibility to psychopathologies such as depression and anxiety disorders (McEwen and Sapolsky 1995; Pittenger and Duman 2008; Sandi 2013).

Neuroplasticity mechanisms play a central role in the brain's resistance to the neurotoxic effects of stress (McEwen 2016; Russo et al. 2012). One of the most important regulators of these mechanisms is brain‐derived neurotrophic factor (BDNF), a protein crucial for synaptic plasticity, neurogenesis, and neuronal survival (Miranda et al. 2019). BDNF exerts these effects largely by binding to its specific receptor, tropomyosin receptor kinase B (TrkB), and is found in high concentrations in regions critical for learning, memory, and executive functions, such as the hippocampus and prefrontal cortex (Bayraktar 2020; B. Lu et al. 2014; Y. Lu et al. 2008). It is essential for the initiation and maintenance of long‐term potentiation (LTP), which forms the molecular basis of learning, making BDNF a key player in the biological infrastructure of cognitive functions (Bekinschtein et al. 2014).

In parallel with this critical role, BDNF levels are highly sensitive to stress. Research shows that chronic stress and increased glucocorticoid levels directly suppress BDNF gene expression, negatively affecting learning and memory processes and paving the way for the onset of depressive symptoms (Bayraktar 2019; Phillips 2017; Sen et al. 2008). However, BDNF levels are not static and can be positively regulated by factors such as physical exercise and enriched environmental stimuli (Szuhany et al. 2015). Therefore, BDNF is considered a central biomarker for brain health and psychological resilience, serving as a bridge between negative experiences like stress and positive interventions like exercise (Phillips 2017).

Neuron‐derived neurotrophic factor (NDNF), a more recently characterized member of the neurotrophic factor family, has been identified as a highly selective marker for a specific subtype of inhibitory neurons, neurogliaform cells, in layer 1 (L1), the outermost layer of the neocortex (Abs et al. 2018; Cohen‐Kashi Malina et al. 2021; Webster et al. 2021). These NDNF‐positive interneurons (NDNF‐INs) slowly and prolongedly suppress the dendrites of pyramidal neurons through a method known as “volume transmission” of GABA (Naumann et al. 2025; Shin and Adesnik 2021). This ultimately contributes significantly to cortical function by enabling more efficient and salient processing of sensory information (Cohen‐Kashi Malina et al. 2021).

The functions of NDNF are not limited to the modulation of cortical circuits; it also plays a critical role in developmental processes (Kowalski et al. 2022). It is known to be involved in neuron migration and survival during development, and mutations in the NDNF gene in humans lead to rare genetic diseases such as congenital hypogonadotropic hypogonadism (CHH) due to neuronal migration disorders (Yang et al. 2022). Finally, NDNF expression shows that it is a significant potential biomarker in the investigation of neurodevelopmental disorders and stress‐related pathologies (Kowalski et al. 2022). Therefore, considering NDNF's modulatory role on the GABAergic system and its potential as a biomarker in stress‐related pathologies, it was thought that this factor could hold exploratory value in understanding the neurobiology of stress associated with higher‐level cognitive processes.

At the center of the stress response lies the activation of the hypothalamic‐pituitary‐adrenal (HPA) axis, and the final product of this process is the glucocorticoid cortisol, known as the “stress hormone” (Herman et al. 2016; Smith and Vale 2006). While its short‐term release is vital for the organism to cope with stress by regulating metabolism and adjusting the immune system, the regulation of this system is disrupted in chronic stress situations, and cortisol levels remain persistently high (McEwen 2016). Indeed, it has been extensively documented that long‐term cortisol exposure has neurotoxic effects on brain regions critical for memory, learning, and emotional regulation, such as the hippocampus and prefrontal cortex (Lupien et al. 2018; McEwen and Sapolsky 1995). Ultimately, these effects lay the groundwork for impairments in cognitive functions and an increased susceptibility to psychopathologies like depression (Pittenger and Duman 2008; Sandi 2013).

The stress response itself and its tendency to become chronic are not merely a passive response to external stimuli (Lazarus and Folkman 1984). Metacognitive skills are reported to be associated with high levels of stress (Drigas and Mitsea 2021). How an individual interprets, evaluates, and manages their own thought processes plays a critical role in sustaining this neurotoxic cycle (Wells 2009). According to the metacognitive model, an individual's beliefs about their thoughts are examined under the headings of positive beliefs about the usefulness of rumination and negative beliefs that rumination is uncontrollable and dangerous (Cano‐López et al. 2022). It is particularly noted that negative metacognitive beliefs exhibit a moderately strong relationship with both rumination and depressive symptoms (Petrošanec et al. 2022). At this point, the concept of metacognition, which refers to an individual's awareness and control over their own cognitive processes, emerges as a potential psychological mechanism that could regulate cortisol release (Matthews and Wells 2003; Ochsner and Gross 2005).

While evidence for the clinical efficacy of metacognitive therapies is growing, empirical research linking these abstract psychological constructs with concrete biological markers of stress and neuroplasticity is still in its infancy (Fisher and Wells 2008). This research aims to fill this significant gap in the literature and to unite metacognition, a psychological model, with biological reality. The main purpose of this study is to examine the relationship between metacognitive beliefs and the levels of cortisol, BDNF, and NDNF obtained non‐invasively from saliva. Within this framework, the following hypotheses will be tested: **H1**: *High negative metacognitive beliefs will be positively associated with high basal salivary cortisol levels*. **H2**: *High negative metacognitive beliefs will be negatively associated with low salivary BDNF levels*. **H3**: *High salivary cortisol levels will be negatively associated with low salivary BDNF levels*. **H4**: *The relationship of NDNF levels with these variables will be examined exploratorily due to limited data in the literature*.

## 2 Method

### 2.1 Research Design

This study is a cross‐sectional investigation designed using a correlational survey model, which is a quantitative research method. Within this model, the existence, direction, and strength of the relationship between the study's main variables—metacognitive beliefs and biological markers of stress and neuroplasticity—were examined. The research data were collected during the fall semester of the 2024–2025 academic year at Bayburt University. Statistical methods such as correlation analysis were used to determine the direction and strength of the relationship between variables, and regression analysis was used to examine the predictive power of metacognitive beliefs on biomarker levels. Consistent with the cross‐sectional design, these analyses aim not to establish a cause‐and‐effect relationship between the variables but to describe the statistical association between them.

### 2.2 Participants

The study included a total of 60 volunteer university students, 30 female (50%) and 30 male (50%), from various faculties at Bayburt University. The participants' ages ranged from 20 to 25. The sample was formed using convenience sampling, a non‐probability sampling method. Inclusion criteria were being an active student at Bayburt University and volunteering to participate in the study. Exclusion criteria were having any diagnosed psychiatric or endocrine disorder, regularly using medication that could affect the HPA axis or neurotrophic factors (e.g., antidepressants, corticosteroids), and having had an infectious disease within the last month.

### 2.3 Procedure

Ethical approval for the study was obtained from the Bayburt University Ethics Committee (Date: March 12, 2025, No: 120/3), and research permission was secured from the institution. Each volunteer who agreed to participate was first given detailed information about the purpose and process of the research, how the data would be used, and how confidentiality would be ensured, and written consent was obtained from all participants. Participants who signed the consent form were given a survey set that included a demographic information form and the Metacognitions Questionnaire‐30 (MCQ‐30). Immediately following the survey administration, saliva sample collection began. To minimize variability arising from the diurnal rhythm of cortisol levels, all samples were collected between 09:00 a.m. and 10:00 a.m. in the morning. Before sample collection, participants were given clear instructions not to eat or drink anything (except water), smoke, chew gum, or brush their teeth for the last hour. Participants were asked to provide approximately 2 mL of saliva into pre‐labeled sterile sample tubes using the passive drool method. The collected samples were immediately placed in transport containers with ice packs to prevent the degradation of biomarkers. All samples were transported to the relevant laboratory on the same day to be stored at −20°C until analysis.

### 2.4 Data Collection Instruments

#### 2.4.1 Measurement of BDNF and Cortisol Hormone Levels in Saliva

Commercial enzyme‐linked immunosorbent assay (ELISA) kits were used to measure the levels of cortisol, BDNF, and NDNF in the saliva samples collected to assess participants' HPA axis activity and neurotrophic factor levels. For BDNF levels, the human‐specific Human BDNF ELISA Kit (SinoGeneclon, Cat. No: SG‐10200, China) was used; for NDNF levels, the Human NDNF ELISA Kit (SinoGeneclon, Cat. No: SG‐16752, China) was used; and for cortisol levels, the Human Cortisol ELISA Kit (SinoGeneclon, Cat. No: SG‐00111, China) was used. All measurements were performed according to the procedures specified in the manufacturer's catalogs, taking into account an intra‐assay coefficient of variation of 8.0% and an inter‐assay coefficient of variation of 10.0%. The results were evaluated by reading the absorbance values at a wavelength of 450 nm as reported in the kit procedure.

#### 2.4.2 MCQ‐30

To measure participants' metacognitive beliefs, the MCQ‐30, developed by Wells and Cartwright‐Hatton (2004) and adapted into Turkish by Tosun and Irak (2008), was used. The scale consists of five sub‐dimensions: (1) Positive Beliefs, (2) Cognitive Confidence, (3) Uncontrollability and Danger, (4) Cognitive Self‐Consciousness, and (5) Need to Control Thoughts. It is important to note that the “Positive Beliefs” subscale does not measure adaptive or positive thinking but rather dysfunctional beliefs about the supposed benefits of worry and rumination (e.g., “Worrying helps me to be prepared”). Therefore, a high score on this subscale, much like the others, contributes to the overall measure of negative or maladaptive metacognitions, which is the focus of this study. The 30‐item scale offers a 4‐point Likert‐type rating (from “(1) Do not agree at all” to “(4) Agree very much”). In the study by Tosun and Irak (2008), the overall internal consistency coefficient (Cronbach's Alpha) of the Turkish form of the scale was reported as 0.860 (Tosun and Irak 2008).

### 2.5 Data Analyses

The statistical analyses of the data were performed using the SPSS 26 software package. Within the scope of descriptive statistics, continuous variables (age, scale scores, hormone levels) were presented as mean ± standard deviation (SD), while categorical variables (gender) were presented as frequency and percentage (%). The normality of the variables' distribution was assessed by examining the results of the Kolmogorov–Smirnov test and the skewness and kurtosis coefficients. As a generally accepted rule in the literature, skewness and kurtosis values falling within the ± 2.0 range were interpreted as not significantly violating the assumption of normal distribution (George and Mallery 2021). Accordingly, Pearson correlation analysis was used to examine the relationships between normally distributed continuous variables. Additionally, linear regression analysis was performed to determine the strength of the association between metacognitive beliefs (independent variable) and biomarker levels (dependent variable). To test the mediating role of cortisol in the relationship between metacognitive beliefs and BDNF levels, a mediation analysis was conducted using a series of linear regression analyses. The statistical significance level was accepted as *p* < 0.05 for all analyses.

## 3 Result

Descriptive statistics and normality test coefficients for the variables addressed in the study are summarized in Table 1. The skewness and kurtosis coefficients, which were examined to assess the normality of the variables' distribution, were found to be within the accepted literary limits of ± 2.0 for all variables.

**TABLE 1:** Descriptive statistics.

Variable	*N* (Valid)	Mean	SD	Skewness	Kurtosis	Min.	Max.
BDNF	60	5.727	2.411	0.015	−1.371	1.01	9.01
Cortisol	60	2.084	0.166	0.323	−0.130	1.76	2.55
NDNF	60	0.943	0.554	0.345	−1.486	0.24	1.91
Metacognition total	60	76.767	13.578	−0.519	1.402	30.00	108.00
Positive beliefs	60	13.717	4.357	−0.116	−0.89	6.00	23.00
Uncontrollability and D.	60	18.567	4.323	−0.210	−0.284	7.00	27.00
Cognitive confidence	60	13.050	4.212	0.186	−1.121	6.00	20.00
Need to control T.	60	15.700	3.321	−0.365	0.076	6.00	23.00
Cognitive Self‐C.	60	15.733	2.869	−0.916	1.884	5.00	20.00

The participants' mean Metacognition Total score (M = 76.77) indicates an average level, being quite close to 75, the theoretical midpoint of the scale's 30‐120 scoring range. A more detailed picture emerges when the metacognition sub‐dimensions are examined: the sample's mean on the Uncontrollability and Danger sub‐dimension (M = 18.57) is significantly above the theoretical midpoint of 15 for its 6–24 point range. This suggests that the participants' beliefs about their thoughts being uncontrollable and dangerous are above average. Similarly, the Need to Control Thoughts (M = 15.70) and Cognitive Self‐Consciousness (M = 15.73) sub‐dimensions are also slightly above the theoretical midpoint of 15. This suggests that the participants' tendencies to control their thoughts and their level of engagement with their own mental processes are at an average level. In contrast, the means for the Positive Beliefs (M = 13.72) and Cognitive Confidence (M = 13.05) sub‐dimensions fell below the theoretical midpoint. This finding shows that the sample has relatively low beliefs about the usefulness of worrying and a low lack of confidence in cognitive abilities like memory. Regarding the biomarkers, the sample's mean BDNF level was 5.73, the mean cortisol level was 2.08, and the mean NDNF level was 0.94. Statistics according to gender differences are presented in Table 2.

**TABLE 2:** Statistics by gender variable.

Variable	Gender	*N*	Mean	SD
BDNF	Female	30	5,246	2,413
	Male	30	6,207	2,351
Cortisol	Female	30	2,126	0,186
	Male	30	2,040	0,133
NDNF	Female	30	0,892	0,555
	Male	30	0,993	0,557
Metacognition total	Female	30	79,00	13,99
	Male	30	74,53	13,00
Positive beliefs	Female	30	13,97	4,506
	Male	30	13,47	4,264
Uncontrollability and D.	Female	30	19,67	4,459
	Male	30	17,47	3,954
Cognitive confidence	Female	30	13,37	4,634
	Male	30	12,73	3,895
Need to control T.	Female	30	15,73	3,571
	Male	30	15,67	3,110
Cognitive Self‐C.	Female	30	16,27	2,490
	Male	30	15,20	3,155

When the mean values presented in Table 2 are examined, some differences are observed between female and male participants. These differences are statistically significant, particularly in cortisol levels and the “Uncontrollability and Danger” sub‐dimension of the Metacognitions Questionnaire. Women's mean cortisol level (M = 2.126) was found to be statistically significantly higher than men's mean level (M = 2.040) (*t* (58) = 2.070, *p* = 0.043). Similarly, women's mean score on the uncontrollability and danger sub‐dimension (M = 19.67) was also statistically significantly higher than men's (M = 17.47) (*t* (58) = 2.022, *p* = 0.048). For the other variables (BDNF, NDNF, metacognition total score, and other sub‐dimensions), no statistically significant difference was found between the mean scores of women and men (*p* > 0.05).

The results of the Pearson correlation analysis, conducted to determine the direction and strength of the relationships between the variables studied, revealed significant findings that support the study's main hypotheses (See Table 3). According to the analysis results, a statistically significant, moderate‐to‐strong positive relationship was found between metacognitive beliefs and cortisol, a physiological stress marker (*r* = .589, *p* < 0.01). When this relationship was examined at the level of metacognition sub‐dimensions, it was observed that the “Uncontrollability and Danger” belief, in particular, showed a significant association with cortisol levels (*r* = 0.468, *p* < 0.01). When the relationship between stress and neuroplasticity markers was examined, a strong, negative, and highly statistically significant correlation was detected between cortisol levels and BDNF levels (*r* = −0.662, *p* < 0.01). This finding indicates that increased HPA axis activity is strongly associated with a decrease in the levels of BDNF, which supports neuronal health.

**TABLE 3:** Pearson correlation analysis results between variables.

Variable	1	2	3	4	5	6	7	8	9
1. NDNF	1								
2. BDNF	0.571**	1							
3. Cortisol	−0.266*	−0.662**	1						
4. Metacognition total	0.048	−0.300*	0.589**	1					
5. Positive beliefs	0.024	−0.269*	0.452**	0.722**	1				
6. Uncontrollability and D.	0.055	−0.173	0.468**	0.861**	0.545**	1			
7. Cognitive confidence	−0.172	−0.339**	0.368**	0.610**	0.495**	0.394**	1		
8. Need to control T.	0.154	−0.038	0.392**	0.662**	0.261*	0.462**	0.177	1	
9. Cognitive Self‐C.	0.152	−0.125	0.236	0.463**	−0.039	0.452**	−0.049	0.353**	1

*Note: N* = 60. **p < 0.01 and *p < 0.05, (two‐tailed).

When the relationship of metacognitive beliefs with neurotrophic factors was examined, a significant negative correlation was found between the Metacognition Total Score and BDNF (*r* = −0.300, p < .05). This negative relationship was found to be more pronounced, particularly with a low perception of “Cognitive Confidence” (*r* = −0.339, *p* < 0.01). NDNF, examined in line with the study's exploratory objectives, was determined to exhibit a moderate‐to‐strong positive relationship with BDNF (*r* = 0.571, *p* < 0.01) and a weak but significant negative relationship with cortisol (*r* = −0.266, *p* < 0.05). However, it was observed that NDNF did not show a statistically significant relationship with the Metacognition Total Score or any of its sub‐dimensions (*p* > 0.05). These results reveal a complex network of relationships among psychological, endocrine, and neurochemical variables.

The results of the mediation analysis are summarized in Table 4. To test the model, the prerequisites for mediation were examined first.

1. *Total effect (Path c)*: First, the total effect analysis (Step 1) showed that metacognitive beliefs had a significant negative association with BDNF levels (*β* = –0.300, *p* = 0.020).
2. *Path a*: Second, the independent variable (metacognition) showed a significant positive association with the mediator (cortisol) (Step 2/Path a: *β* = 0.589, *p* < 0.001).
3. *Mediator to outcome*: Third, the mediator (cortisol) showed a strong, significant negative association with the outcome variable (BDNF) (*r* = –0.662, *p* < 0.01; see Table 3).

**TABLE 4:** Hierarchical multiple regression analysis for variables associated with BDNF levels.

Model/Path	Predictor(s)	Outcome	*β*	*p*‐value	Model *R^2^*
Step 1: Total effect (Path c)	Metacognition (Total)	BDNF	−0.300*	0.020	0.090
Step 2: IV to Mediator (Path a)	Metacognition (Total)	Cortisol	0.589**	< 0.001	0.347
Step 3: Mediation model		BDNF			0.169
(Path b)	Cortisol		−0.281*	0.024	
(Path c')	Metacognition (Total)		−0.298*	0.017	

*Note*: The table displays the regression paths testing the mediating role of cortisol in the relationship between metacognition and BDNF. Path a result is based on the correlation reported in Table 3, where β equals r in a simple linear regression. Other results are derived from the hierarchical regression output.

**Abbreviations**: *β* = Standardized beta coefficient, IV = Independent Variable.

**p < 0.01 and *p < 0.05.

*Mediation Model (Step 3)*: Finally, the mediation model was tested by including both metacognition and cortisol as simultaneous predictors of BDNF. The results showed that the indirect path through cortisol was significant (Path b: *β* = –0.281, *p* = 0.024). Crucially, the direct effect of metacognition on BDNF (Path c') also remained statistically significant after controlling for cortisol (β = –0.298, *p* = 0.017).

**FIGURE 1:**
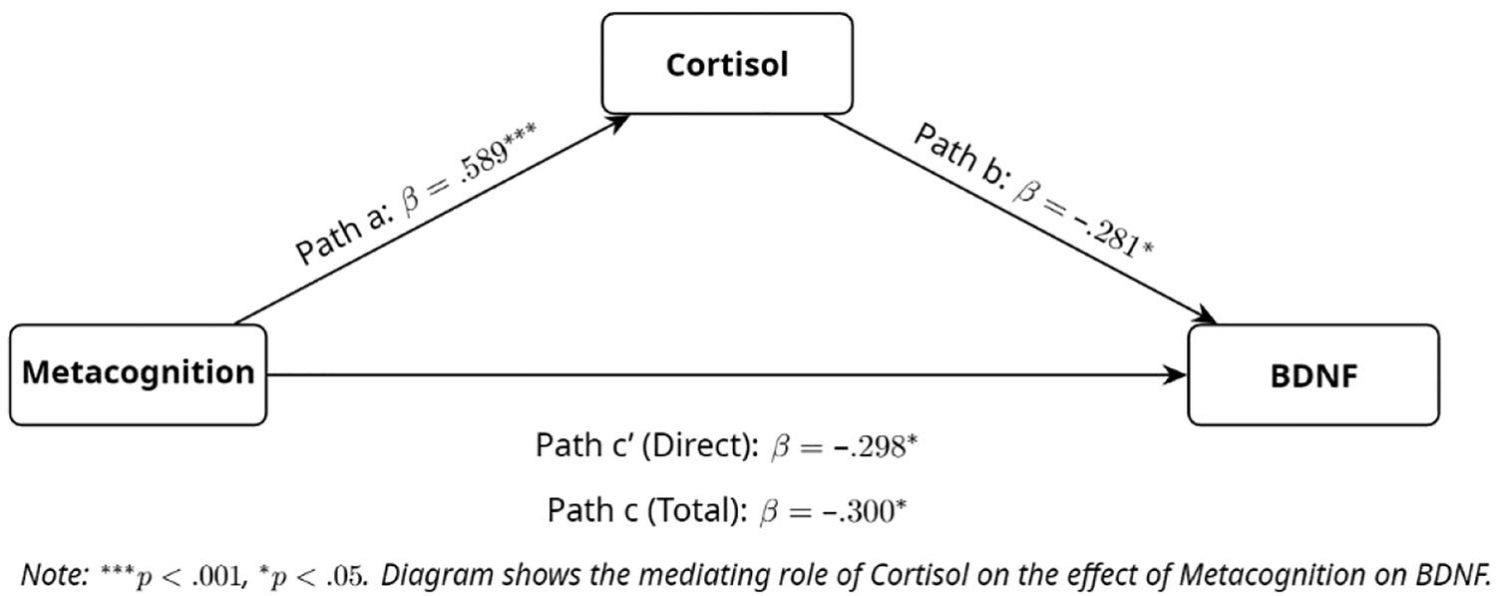
The mediation model between metacognition, cortisol, and BDNF.

This pattern, where both the indirect effect and the remaining direct effect are significant, is consistent with a partial mediation model. To further quantify this, the proportion of the total effect mediated by cortisol was calculated (Indirect Effect / Total Effect), revealing that the indirect pathway accounted for approximately 55.1% of the total relationship. This finding suggests that dysfunctional metacognitive beliefs are associated with lower BDNF levels through two distinct pathways: a significant direct association and a significant indirect association partially mediated by cortisol. This partial mediation model is visualized in Figure 1.

A multiple linear regression analysis was also conducted to examine the associations of biological markers with NDNF levels. As shown in Table 5, the overall model was statistically significant, *F* (2, 57) = 15.279, *p* < 0.001, with the two variables together accounting for approximately 35% of the variance in NDNF levels (*R^2^* = 0.349). When the individual variables were examined, BDNF was found to have a statistically significant and strong positive association with NDNF levels (*β* = 0.704, *p* < 0.001). However, after controlling for the effect of BDNF, cortisol was not found to have a significant association with NDNF (*p* = 0.166).

**TABLE 5:** Multiple linear regression analysis results for biological markers associated with NDNF levels.

Predictor	B	Ss	*β*	*t*	*p*
(Constant)	−2.300	1.500		−1.533	0.131
Cortisol	0.922	0.658	0.200	1.402	0.166
BDNF	0.191	0.039	0.704	4.936	**< 0.001**
Model summary	***R^2^* = 0.349 F (2, 57) = 15.279 *p* < 0.001**				

Dependent Variable: NDNF.

## 4 Discussion

This study was designed to investigate the relationship between an abstract psychological construct, such as metacognitive beliefs, and concrete biological markers of stress and neuroplasticity: salivary cortisol, BDNF, and NDNF levels. The research findings largely supported the main hypotheses by revealing a significant network of relationships among psychological, endocrine, and neurochemical systems. The most fundamental finding was that dysfunctional metacognitive beliefs are associated with increased cortisol and decreased BDNF levels. Regression analyses took this relationship a step further, providing strong evidence that suggests cortisol may play a potential mediating role in the relationship between negative metacognitions and BDNF. NDNF, which was examined exploratorily, exhibited a strong biological connection with BDNF but did not show a direct relationship with metacognitive processes. These results offer important data on the biological foundations of the metacognitive model and provide a holistic perspective on the neurobiology of stress.

Our study has revealed that negative metacognitive beliefs are significantly associated with an increase in the levels of cortisol, the primary stress output of the HPA axis. The positive correlation between beliefs that “thoughts are uncontrollable and dangerous” and high cortisol levels can be interpreted as a biological reflection of the metacognitive model. This finding indicates that an individual's perception of their own mental processes can directly modulate the physiological stress response. There is evidence in the literature that stress can impair metacognitive accuracy, meaning it can weaken an individual's judgments about their own performance. Our study, however, illuminates the other side of this relationship: dysfunctional metacognitive beliefs themselves can act as an internal stressor that activates the stress system. The Self‐Regulatory Executive Function (S‐REF) model, proposed by Wells and Matthews, also suggests that such metacognitive beliefs underlie cyclical thought patterns like worry and rumination and perpetuate psychopathology. In this context, our findings make a significant contribution to the literature by pointing to a physiological mechanism of this model, namely the activation of the HPA axis.

The clearest and statistically most powerful finding of the study is the pronounced negative relationship between cortisol and BDNF. Increased cortisol levels are strongly associated with a decrease in BDNF, a critical molecule for neuronal health and plasticity. This result is in complete agreement with numerous studies showing that chronic stress reduces BDNF expression and, through this pathway, can lead to neuronal atrophy in brain regions like the hippocampus. One of the most original contributions of this study is its elucidation of the complex relationship between metacognition, cortisol, and BDNF. The regression analysis revealed that cortisol plays the role of a partial mediator in this relationship. The findings indicate that dysfunctional metacognitive beliefs are associated with BDNF levels through two distinct pathways: first, a significant direct effect that is independent of cortisol, and second, a significant indirect effect that occurs through cortisol levels. This suggests that dysfunctional metacognitions may impact BDNF not only by triggering the HPA axis but also through other mechanisms not accounted for by cortisol.

This finding offers a concrete hypothesis on how psychological interventions, such as metacognitive therapy, might lead to biological improvement. When dysfunctional beliefs are modified through therapy, it is possible that not only does the chronic pressure on the HPA axis decrease, allowing cortisol levels to normalize, but the direct negative association of these beliefs with BDNF may also be alleviated. This suggests that therapy could provide a dual benefit in protecting brain health.

An important nuance in our findings relates to the “Positive Beliefs” subscale of the MCQ‐30. Our results indicated a negative correlation between this subscale and BDNF levels, which might seem counterintuitive. However, within the metacognitive model, “Positive Beliefs” refer to maladaptive beliefs about the utility of worry and rumination. A high score reflects a stronger conviction that these negative thought patterns are useful, thereby promoting more persistent cognitive stress. This chronic internal stress is consistent with the activation of the HPA axis and, consequently, the suppression of neuroprotective factors like BDNF. Thus, the inclusion of this subscale in the total metacognition score does not dilute the focus on ‘negative metacognitions’; rather, it enriches it by capturing a key mechanism that initiates and sustains the cycle of worry and rumination.

The strong negative relationship between cortisol and BDNF (r = ‐.662) identified in our study is consistent with the predominant view in the literature that supports the neurotrophic hypothesis (Phillips 2017; Pittenger and Duman 2008). However, in a study conducted by Sözeri‐Varma et al. (2012) on 30 patients diagnosed with major depressive disorder, no significant relationship was found between serum BDNF and cortisol levels. The basis for this difference between our findings may lie in the nature of the psychological constructs measured. While the study by Sözeri‐Varma et al. focused on a clinical diagnosis like major depressive disorder, our study examines a more fundamental mechanism—dysfunctional metacognitive beliefs—that may lay the groundwork for such disorders. Therefore, our findings may be tracing a biological marker related to vulnerability to pathology rather than the pathology itself. This aligns with the broader neurotrophic hypothesis of depression, which posits that reduced BDNF levels, particularly in response to stress, represent a key biological vulnerability that increases an individual's risk for developing mood and anxiety disorders (Pardossi et al. 2024; Nazareth 2021; Phillips 2017; Neto et al. 2011; Kotan et al. 2009).

Nevertheless, both studies present a common theme that high cortisol levels are associated with negative outcomes. Sözeri‐Varma et al. (2012) found that high cortisol levels were associated with impaired performance on the Stroop test, that is, a weakening of attentional processes. Our study, on the other hand, linked high cortisol with both psychological and neurochemical adversities, such as dysfunctional metacognitive beliefs and low BDNF levels. The fact that our study was conducted on a non‐clinical sample of healthy young adults, rather than being a limitation, offers an opportunity to understand the early‐stage biological changes and vulnerability mechanisms that precede the development of stress‐related disorders. Our findings show that even before psychopathology emerges, dysfunctional thought patterns leave measurable traces on the HPA axis and neuroplasticity.

NDNF, examined in line with our study's exploratory objectives, did not show a direct relationship with metacognitive beliefs but did exhibit a strong positive correlation with BDNF. Regression analyses revealed that the primary biomarker significantly associated with NDNF levels was BDNF rather than cortisol. This suggests that these two neurotrophic factors are regulated through common or interrelated biological pathways. When NDNF's role in the literature is reviewed, it is known that this molecule plays a critical role in fundamental neurodevelopmental processes such as neuron migration, growth, and axon development (Hassani et al. 2025; Kuang et al. 2010). The absence of a significant relationship between metacognition and NDNF in our study suggests that NDNF's response to stress may be modulated at a more fundamental and physiological level (such as basic neuronal integrity) via BDNF, rather than through high‐level cognitive processes like metacognition. This finding indicates that further research is needed to elucidate the potentially different roles of NDNF and BDNF in stress response mechanisms.

Furthermore, our study, conducted on a non‐clinical young adult (university student) sample, showed that dysfunctional metacognitions are associated with increased cortisol and decreased BDNF. These findings support the general consensus that psychosocial stress activates the HPA axis, thereby suppressing the BDNF system (Agrimi et al. 2024). However, an interesting picture emerges when compared with similar studies conducted on university students. For example, a study by Ballestar‐Tarín et al. (2024), also on university students, found that depressive symptoms were associated with higher salivary BDNF levels. Moreover, while Ballestar‐Tarín's study found no relationship between cortisol levels and depressive symptoms, the strong relationship of cortisol with both metacognition and BDNF in our study suggests that the metacognitive mechanism we examined may have a more direct link to HPA axis activation. These differences emphasize that the effect of stress on biomarkers can vary depending on the stage of the process and the specific psychological constructs being measured.

### 4.1 Limitations

Some limitations should be considered when interpreting the findings of this study. First, the sample consists of a relatively small group of 60 individuals and comprises university students selected through convenience sampling. This situation limits the generalizability of the results to broader populations. The study's cross‐sectional design does not permit the interpretation of the identified relationships between variables as cause‐and‐effect. For example, it cannot be determined with this design whether negative metacognitions increase cortisol or if high cortisol leads to such thought patterns. Furthermore, it should be noted as a potential limitation that the data collection was carried out during the fall semester, which is part of the academic calendar for university students. It has been shown in the literature that academic stress can affect BDNF expression. Therefore, the possibility that some of our findings may have been influenced by the academic stress participants were exposed to during that period cannot be disregarded. Furthermore, our study focused on metacognitive beliefs, which can also be affected by transient stress, but did not assess more stable personality traits using appropriately standardized instruments; this means that more fundamental psychological structures underlying the findings may have been overlooked.

One of the most significant methodological limitations is that cortisol levels were measured at only a single time point in the morning. Considering the diurnal rhythm of cortisol, assessing the delta between the morning peak (zenith) and the evening low (nadir) could have more accurately reflected HPA axis dysregulation. Therefore, our findings are limited to basal cortisol levels and do not reflect potential disruptions in the cortisol rhythm. Similarly, morning salivary cortisol levels can also be affected by sleep duration and quality. In our study, data regarding the sleep patterns of the participants were not collected, and it is likely that this may have introduced some variance in the cortisol measurements.

Additionally, metacognitive beliefs were measured with a self‐report scale, which may be susceptible to biases such as social desirability. The collection of both metacognitive beliefs and demographic data through the same survey method introduces the possibility that the findings could be affected by common method variance. Finally, this study focused solely on the neuroendocrine (HPA axis) dimension of stress; a psychophysiological analysis aimed at assessing the subjects' sympathovagal balance was not performed. This overlooks the potential effects of metacognitions on the autonomic nervous system, thereby limiting the presentation of a holistic picture of the mind‐body interaction.

### 4.2 Recommendations

For future studies, the use of longitudinal or experimental designs is recommended to test the causality of the findings. For instance, studies that measure these biomarkers before and after an intervention, such as metacognitive therapy, could elucidate this mechanism more clearly. Moreover, future research examining the cortisol response to an acute stressor, like the Trier Social Stress Test (TSST), could clarify whether metacognition is more related to basal cortisol levels or to stress reactivity. Furthermore, it is recommended that future studies collect cortisol samples at a minimum of two time points, morning (zenith) and evening (nadir), to calculate the delta between them, which would provide a more dynamic and valid assessment of HPA axis activity. On a psychological level, future research should incorporate not only metacognitive beliefs but also stable personality traits, such as neuroticism, into the model to help understand the more enduring structures underlying stress vulnerability. On a biological level, it is important to expand the scope of research beyond the HPA axis to also include the autonomic nervous system. Psychophysiological analyses, such as heart rate variability (HRV), aimed at assessing the subjects' sympathovagal balance would offer a more holistic picture of the effects of metacognitions on somatic stress responses. Studies conducted with larger and more diverse samples will increase the generalizability of the findings. Our findings also suggest that therapeutic interventions focusing on metacognitive processes in the treatment of stress‐related psychopathologies may have a biological basis. It should be considered that stress management programs may not only provide psychological relief but also create positive effects on brain health by protecting neurotrophic factors like BDNF.

## 5 Conclusion

In conclusion, this study has shown that there is a significant relationship between metacognitive beliefs, HPA axis activity, and neurotrophic factors. Strong evidence has been presented showing that negative metacognitive beliefs are associated with low BDNF levels through both a direct pathway and an indirect pathway via cortisol. The findings support a partial mediation role for cortisol in this relationship. These findings reveal the concrete effects of psychological processes on biological health and emphasize the importance of metacognitive interventions in the prevention and treatment of stress‐related disorders. Future research supporting these findings with longitudinal and experimental studies will further deepen our understanding in this area.

## Author Contributions

S.O. and B.B. designed the study. S.O., B.B., and F.T.K. collected data. S.O. analyzed the data. B.B. and F.T.K. prepared the draft plan. All authors contributed to writing the manuscript. All authors read and approved the final manuscript.

## Funding

The authors have nothing to report.

## Ethics Statement

The research was approved by the Bayburt University Research Ethics Committee (12.03.2025/Decision no: 120/3). The researchers were informed about the study and written/verbal consent was obtained from the participants in accordance with the Declaration of Helsinki before data and samples were collected. All methods were carried out in accordance with the relevant guidelines and regulations.

## Conflicts of Interest

The authors declare no conflicts of interest.

## Data Availability

The corresponding author upon reasonable request will provide data supporting the findings of this study.
